# The use of pediatric short-stay observation in Italy

**DOI:** 10.1186/s13052-023-01441-8

**Published:** 2023-03-21

**Authors:** Luciano Pinto, Sonia Bianchini, Maria Antonietta Barbieri, Gabriella Cherchi, Andrea Miceli, Maria Pia Mirauda, Valeria Spica Russotto, Irene Raffaldi, Tiziana Zangardi, Domenico Perri, Rino Agostiniani, Simone Rugolotto, Fabio Cardinale, Stefania Zampogna, Annamaria Staiano

**Affiliations:** 1 Italian Society of Pediatric Emergency Medicine, Via Nevio 60, 80122 Naples, Italy; 2 Department of Pediatrics, San Carlo Borromeo Hospital, Via Pio II 3, 20153 Milan, Italy; 3 Emergency Department, Pediatric Hospital Bambino Gesù, Via Torre di Palidoro, 00050 Fiumicino, Rome, Italy; 4 Emergency Department, SsD Pediatric Emergency Medicine, ARNAS G Brotzu Hospital, Piazzale Alessandro Ricchi 1, 09047 Selargius, Cagliari, Italy; 5 Department of Pediatrics, Civil Hospital of Pavullo, Via Suore SBG Cottolengo 1, 41026 Pavullo nel Frignano, Modena, Italy; 6 Department of Pediatrics, San Carlo Hospital, Via Potito Petrone, 85100 Potenza, Italy; 7 Del Ponte Hospital, Department of Pediatrics, ASST Settelaghi, Via Del Ponte 19, 21100 Varese, Italy; 8 Pediatric Emergency Department, Regina Margherita Hospital, Città della Salute e della Scienza di Torino, Piazza Polonia 94, 10126 Turin, Italy; 9 Department of Women’s and Children’s Health, University of Padua, 35128 Padua, Italy; 10 Department of Pediatrics, San Giuseppe Moscati Hospital, Via Antonio Gramsci 1, 81031 Aversa, Italy; 11 Department of Pediatrics and Neonatology, San Jacopo Hospital, Via Ciliegiole 97, 51100 Pistoia, Italy; 12 Department of Pediatrics, Santa Maria della Misericordia Hospital, Viale Tre Martiri 140, 45100 Rovigo, Italy; 13 Department of Pediatrics and Emergency, Pediatric Allergy and Pulmunology Unit, Azienda Ospedaliera-Universitaria “Consortium-Policlinico” University Hospital, Pediatric Hospital Giovanni XXIII, Via Amendola 207, 70123 Bari, Italy; 14 Department of Pediatrics, San Giovanni di Dio Hospital, Via Bologna 88900, Crotone, Italy; 15 Department of Translational Medical Sciences - Section of Pediatric, University Federico II, Via Pansini 5, 80131 Naples, Italy

**Keywords:** Observation, Short-Stay Observation, Pediatrics, Emergency Department, Hospital, Triage, Survey, Guidelines

## Abstract

**Background:**

In Italy, the State Regions Conference on 1^st^ August 2019 approved the Guidelines for Short-Stay Observation (SSO). At the beginning of 2022, the main Scientific Societies of the pediatric hospital emergency-urgency area launched a national survey to identify the extent to which these national guidelines had been adopted in the emergency rooms and pediatric wards of the Italian Regions.

**Methods:**

A survey has been widespread, among Pediatric Wards and Pediatric Emergency Departments (EDs), using both a paper questionnaire and a link to a database on Google Drive, for those who preferred to fill it directly online. Those who did not spontaneously answer, where directly contacted, via email and/or through a phone call and invited to participate. The data collected have been: age of managed children, presence of triage, presence of Sub-intensive Care Unit and Intensive Care Unit and special questions about Pediatric SSO, availability of training courses for workers, number of ED access in the last 4 years.

**Results:**

This survey is still ongoing, without a definite deadline, so we presented the preliminary data.

Currently, 8/20 Regions have not yet adopted the Guidelines. Till 02 January 2023, data from 253 hospitals were collected. There are currently 180/253 active Pediatric SSO (71.03% of the Hospitals). There are not active SSO in 33.27% of first level ED, in 19.35% of second level ED and in 33.66% of General Hospitals with Pediatric Wards. Active SSO are located mainly (75.97%) within Pediatric Wards. At the moment, the survey has been completed in 16 Regions: in the 8 Regions which are using guidelines, pediatric SSOs are active in all the second level ED (compared to 60.87% of the other 8 regions), in the 91.66% of first level ED (compared to the 33.3%), and in the 97.1% of General Hospitals (compared to 33.3%), with a statistically significance (*p* < 0.0001).

The territorial analysis of these 16 regions highlighted geographical differences in the percentage of SSOs active: 35.22% are active in hospitals in Southern Italy, 88.64% in Central Italy and 91.67% in those of the North.

**Conclusions:**

The delay in adopting specific guidelines negatively influences activation of pediatric SSOs in hospital system and prevents the adjustment of welfare level to new needs. To facilitate the activation of SSOs in hospitals, it is also necessary to guarantee adequate economic recognition. It is essential to implement public interventions to overcome the current inequalities in the interest of children and their families: the current delay seriously penalizes emergency pediatric hospital care, especially in the southern Italian Regions.

## Background

There is a growing awareness that hospitalization is not always necessary for the majority of the children who come to the emergency room, with an acute illness. An alternative route is represented by the pediatric Short-Stay Observation (SSO), a specific area of the hospitals used to diagnose and/or treat a medical situation, in a short well-defined period of time (generally less than 24 or 48 h). The word “observation” suggests a frequent evaluation of the patients to monitor disease progression or response to therapy [1].

In Italy, as declared by the 1996 Status – Region Act, SSO were a specific part of the Emergency Department (ED) [2].

The 2005 SIMEUP Consensus Meeting has defined the structural and organizational standards of pediatric SSO, which should be performed in functional areas related to the ED or inside the Pediatric Wards [3]. Following these indications, different regions have issued measures to institutionalize pediatric SSO inside their hospital network.

The work of Longhi et al. analyzed the ED activities, focusing on the SSO service, which the authors defined as a potential extremely useful tool in decreasing and optimizing pediatric hospital admissions. Data reported in this survey, from 237 of the 624 active Pediatric Wards, evidenced that in the 2010–2011 period, 66% of the ED had a pediatric SSO: 80% of the structure in North Italy, 67% of those in the Center of Italy and 43% of those in South Italy, with statistically significant differences between North and South regions (*p* < 0.001) and between Center and South regions (*p* = 0.025) [4].

The 2015 Ministerial Decree (M. D. n. 70) established that all hospitals, which have an ED, starting from those which have catchment area of 80,000 to 150,000 inhabitants, should have some beds dedicated to SSO [5].

The Status – Region Accordance n.248 (21^st^ December 2017) [6] has clarified in the “Guidelines for promoting and bettering of quality, security and appropriateness of welfare interventions in pediatric and adolescent areas – 10 guides of action” the following points:

- The main action, to be done to improve the response of welfare network, is to guarantee a full-time pediatric assistance (24 h /24 h) in all hospitals with a pediatric ward, as well as ED activity and SSO;
- SSO, which generally lasts 24 h and only occasionally 36 h, should be organized in all pediatric hospitals with careful evaluation of resources and obtainable results in terms of hospitalization reduction, rationalization of welfare pathways and costs restrains.

The Status – Region Accordance n. 143 (1^st^ August 2019), together with National Guidelines of Hospital Triage and National Guidelines on plan to manage Overcrowding in ED, has approved National Guidelines on SSO, which is the first Italian public act on this important topic [7]. The paper asserted that pediatric SSO, in the absence of pediatric ED, could be done inside the Pediatric Ward, if the pediatric ward is able to perform a self-triage and an autonomous discharge: in this case, specific beds and spaces for SSO should be identified, taking into account also the presence of a caregiver for each patient. Inclusion and exclusion criteria for SSO patients are reported in the Guidelines.

All Italian Regions accepted to promote the contents of Accordance within 6 months from its approval and the Minister of Health was committed to create a worktable within 3 months, to find a method to determine welfare standard costs in SSO and its related way of payment: more than three years have passed, and no information about this activity has been disclosed.

### Aim of the study

The Italian Society of Pediatrics (SIP), the Italian Society of Pediatric Emergency Medicine (SIMEUP) and the Italian Society of Hospital Pediatrician (SIPO) promoted a national survey to know if the national guidelines on Hospital Triage and SSO in EDs and Pediatric Wards among Italian Regions have been adopted. The second aim was to highlight features of different Pediatric EDs, Pediatric Wards, and SSO in Italian Hospitals.

## Methods

A survey has been created, using both a paper questionnaire and the link to a database on Google Drive. The data collected have been: age of managed children, presence of triage, presence of Sub-intensive Care Unit and Intensive Care Unit and special questions about Pediatric SSO, availability of training courses for workers, number of ED access in the last 4 years.

National Presidents of Scientific Society were invited to widespread the questionnaire among Directors of Pediatric ED and Pediatric Wards.

Those who did not spontaneously answer, where then directly contacted, via email and/or through a phone call, and invited to fill in the questionnaire and to provide requested information.

In order to reduce potential missed data, by consulting the Ministry of Health list about hospital emergency network (2019 and 2022 editions) and through specific research about hospital pediatric welfare network of each single region, every hospital with ED in each region has been contacted in order to verify the availability of a Pediatric ED and / or a Pediatric Ward [8].

Collected data have been analyzed using Microsoft Excel Pivot and MedCalc statistical software (https://www.medcalc.org/); to best highlight our results, we reported also the data by each single region, to allow Regional President to evaluate the state of the art of pediatric hospital welfare and specifically about SSO.

### Primary results

This survey is still ongoing, without a definite deadline, so we presented the preliminary data, collected at 1^st^ January 2023.

Currently, data collection has been completed in 16 regions (Abruzzo, Basilicata, Calabria, Campania, Emilia-Romagna, Friuli-Venezia-Giulia, Lazio, Liguria, Marche, Molise, Puglia, Sardinia, Tuscany, Umbria, Valle d’Aosta, Veneto), where at 1^st^ January 2022, there were 5,995,349 inhabitants aged under 17 years (equal to 65.16% of the 9,200,287 younger inhabitants surveyed in Italy). Data are still being collected in the other regions (Piedmont, Lombardy, A.P. of Bolzano and Trento, Sicily): in these regions at 1^st^ January 2022, 3,204,938 younger inhabitants were reported (34.84% of the younger surveyed in Italy).

The hospitals have been divided, according to their features, into General Hospitals, first and second level EDs and Pediatric Hospitals.

According to the current legislation [5], General Hospitals could not have a specific Pediatric Ward, whereas they should always be present in first level EDs, which are the Spoke of pediatric hospital emergency network, and in second level ED, which represent the Hub.

Out of the 253 received surveys, there are 180 Pediatric SSOs in activity (71.15% of the Hospitals). There are no active Pediatric SSO in the 33.67% of first level EDs, in the 19.35% of second level EDs, and in the 33.27% of General Hospitals with Pediatric Wards (Table 1).

**Table 1:** Active pediatric SSOs classified by the type of hospitals

Type of Hospital	Total	Active SSO	
		N	%
EDs 1st level	98	65	66.33
EDs 2nd level	62	50	80.65
PH	16	14	87.50
GH	77	51	66.23
Total	253	180	71.15

Active SSO are mainly located (76.11%) within Pediatric Wards (Table 2).

**Table 2:** Dislocation of pediatric SSOs

Type of hospital	General DEA	Pediatric DEA	Pediatric ward	Total SSOs
EDs 1^st^ level	1	7	57	65
EDs 2^nd^ level		16	34	50
PH		14		14
GH		5	46	51
**Total**	1	42	137	180

Percentage of active SSO is lower in regions in which national guidelines have not yet been adopted and in regions, such as Lombardy, in which they have been recently adopted (Table 3).

**Table 3:** Dislocation of active SSOs in the different Italian Regions

**Regions**	**EDs **^**1st**^** level**			**EDs 2**^**nd**^** level**			**PH**			**GH**			**Total**	**SSOs active**		**Adopted guidelines**	**Regional survey**
	**No**	**Yes**	**Total**	**No**	**Yes**	**Total**	**No**	**Yes**	**Total**	**No**	**Yes**	**Total**		**N°**	**(%)**		
Abruzzo	2	1	**3**		1	**1**				3	2	**5**	**9**	4	44.44	No	Completed
Basilicata	1		**1**		1	**1**				1	2	**3**	**5**	3	60.00	No	Completed
Calabria	6	2	**8**		3	**3**					1	**1**	**12**	6	50.00	No	Completed
Campania	6	2	**8**	3	5	**8**		1	**1**	8	1	**9**	**26**	9	34.62	No	Completed
E. Romagna	1	2	**3**		9	**9**		1	**1**		5	**5**	**18**	17	94.44	Yes	Completed
FVG	1	3	**4**		2	**2**		1	**1**		2	**2**	**9**	8	88.89	Yes	Completed
Lazio	6	8	**14**	1	4	**5**	2^a^		**2**		1	**1**	**22**	13	59.09	No	Completed
Liguria	1	3	**4**		1	**1**		1	**1**				**6**	5	83.33	Yes	Completed
Lombardy		1	**1**	2	5	**7**		3	**3**	2	3	**5**	**16**	12	75.00	Yes	Not completed
Marche		5	**5**					1	**1**		4	**4**	**10**	10	100.00	Yes	Completed
Molise	1		**1**							2		**2**	**3**	0	0.00	No	Completed
PA Bolzano		2	**2**		1	**1**					1	**1**	**4**	4	100.00	Yes	Not completed
PA Trento					1	**1**							**1**	1	100.00	Yes	Not completed
Piedmont		12	**12**		4	**4**		2	**2**	1	2	**3**	**21**	20	95.24	Yes	Not completed
Puglia	7	1	**8**	3		**3**		1	**1**	6	2	**8**	**20**	4	20.00	No	Completed
Sardinia	1	1	**2**	2		**2**				2	2	**4**	**8**	3	37.50	No	Completed
Sicily		2	**2**	1	1	**2**							**4**	3	75.00	No	Not completed
Tuscany		9	**9**		5	**5**		1	**1**	1	6	**7**	**22**	21	95.45	Yes	Completed
Umbria		3	**3**		3	**3**							**6**	6	100.00	Yes	Completed
Valle d'Aosta											1	1	**1**	1	100.00	Yes	Completed
Veneto		8	**8**		4	**4**		2	**2**		16	16	30	30	100.00	Yes	Completed
**Total**	**33**	**65**	**98**	**11**	**50**	**61**	**2**	**14**	**16**	**26**	**51**	**77**	**253**	**180**	**71.15**		

^a^In these 2 Pediatric Hospitals, the Short-Stay Observation has not yet been activated due to lack of space

**Fig. 1:**
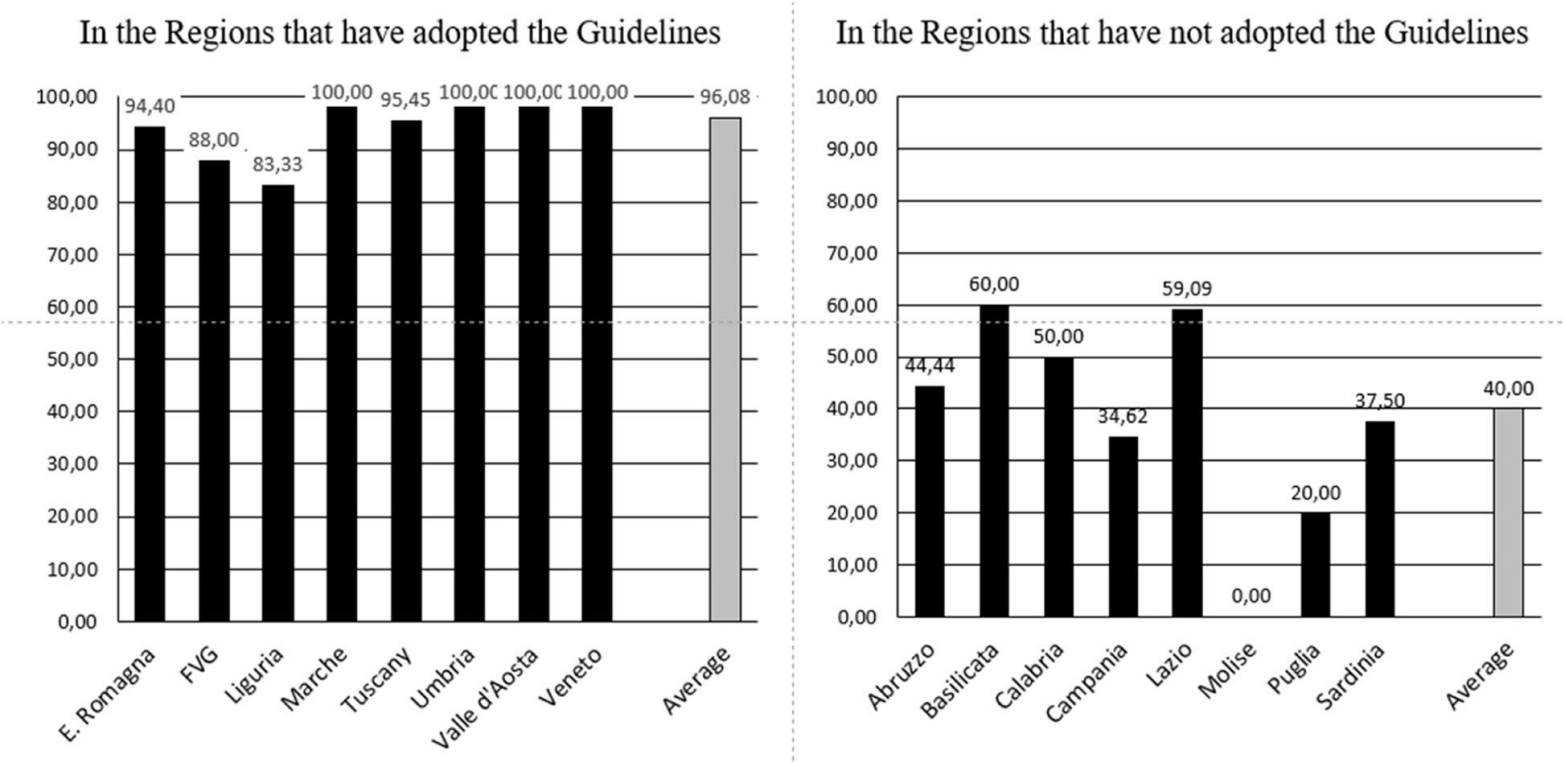
Percentage (%) of active pediatric SSOs among all hospitals in the 16 Regions which have completed the survey

Statistically significant differences have been highlighted among the 16 regions in which the survey collection has been completed, independently from the features of hospital considered (first level EDs, second level EDs, General Hospitals) (Fig. 1):

- The regions of Emilia-Romagna, Friuli-Venezia-Giulia, Liguria, Marche, Tuscany, Umbria, Valle d’Aosta, Veneto have adopted guidelines; in these regions, in which altogether live 2.706.425 subjects less than 17 years old, there are 102 Hospital with Pediatric ED and / or Pediatric Wards, and the percentage of active SSOs is 96,08%.
- The regions of Abruzzo, Basilicata, Calabria, Campania, Lazio, Molise, Puglia and Sardinia have not yet adopted guidelines: here there are 3,288,924 subjects aged less than 17 years, there are 105 Hospital with Pediatric ED and / or Pediatric Wards and the percentage of active SSOs is 40.00%

In the 8 regions which are following guidelines, SSOs are active in all the second level EDs (compared to 60.87% of the other 8 regions), in the 91.66% of first level EDs (compared to the 33.3%), and in the 97.1% of General Hospitals (compared to 33.3%) (Table 4). Differences, calculated with McNemar test, result statistically significant: *p* < 0.0001 (Table 5).

**Table 4:** Distribution of SSOs on the base of adoption of national guidelines in the 16 Regions which have completed the survey

Type of hospital	Active SSOs			
	Adopted guidelines		Guidelines NOT adopted	
	No	Yes	No	Yes
EDs 1^st^ level	3 (8.33%)	33 (91.66%)	30 (66.66%)	15 (33.33%)
EDs 2^nd^ level		24 (100%)	9 (39.13%)	14 (60.87%)
PH		8 (100%)	2 (50%)	2 (50%)
GH	1 (2.9%)	34 (97.1%)	22 (66.6%)	11 (33.3%)

**Table 5:** Data analysis using the McNemar Test

Active pediatric SSOs	Adopted guidelines	
	Yes	No
Yes	98	42
No	4	63

*p*: < 0.0001

**Fig. 2:**
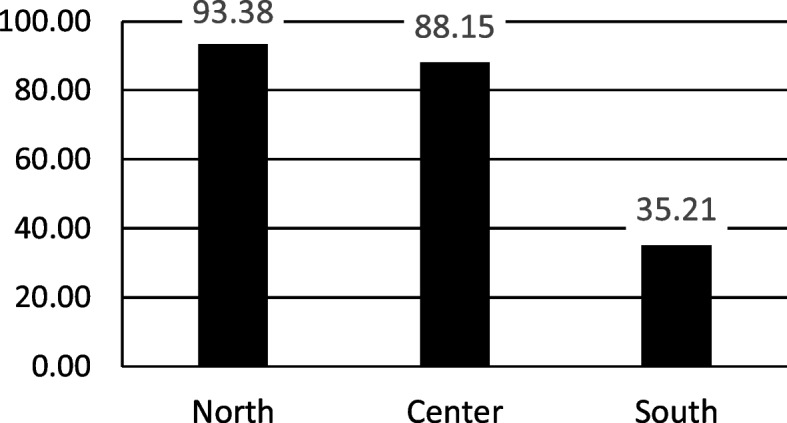
Percentage (%) of active SSOs among the hospitals of the 16 Regions of Italy by geographical areas

The territorial analysis of these 16 regions highlighted significant geographical differences in the percentage of active SSOs in the hospitals of the 3 areas of Italy: North (Emilia-Romagna, Friuli-Venezia-Giulia, Liguria, Valle d’Aosta, Veneto) 93.38%; Center (Lazio, Marche, Umbria, Tuscany) 88.15%; South (Abruzzo, Basilicata, Calabria, Campania, Molise, Puglia, Sardinia) 35.21%. (Fig. 2).

Noteworthy, in Molise there are not pediatric SSO, although there are 3 hospitals with a Pediatric Ward, 2 of which are first level ED; moreover, in some regions of Southern Italy, there are some active SSOs although regions have not yet adopted guidelines and do not provide any remuneration. Furthermore, also in regions in which SSO has been adopted, there are first level ED with Pediatric Wards without active SSOs.

## Discussion

In Italy the regions are responsible themselves of the organization and delivery of services through their Regional Healthcare Systems; so, in our Country there are 21 different health systems.

Not all regions, and so not all their hospitals, have adopted national guidelines, whose goal was improving ED functionality, allowing the discharge of patients affected by acute disease which could be rapidly resolved directly from the ED, without resorting to hospitalization.

The data collected showed important differences in adopting national guidelines on SSO, confirming what already reported by Longhi et al [4]. The delay in adopting specific guidelines negatively influences activation of SSOs in hospital system and prevents the adjustment of welfare level to new needs.

We want to highlighted that the collection of data has not been simple, as documented by the ongoing state of the survey, as. Our data, confirmed the percentage of participation reported by the group of Longhi et al., because Emergency Care Directors are overly busy and also due to the well-known deficiency of hospital and emergency-care pediatrician we nowadays suffer in Italy [4].

It should also be emphasized that there is no news from the technical table to determine the remuneration of the SSOs, despite the commitment made by the Ministry of Health to activate it within three months from the date of approval of the Guidelines. Currently the remuneration is very low, inadequate compared to the needs of intensive care: an adequate remuneration could lead several hospitals to activate pediatric SSOs even in the absence of resolutions from their Regions.

## Conclusion

To our knowledge, this work represents the most updated survey on Italian situation regarding activation and features of SSO.

The delay in the activation of the pediatric SSOs forces minors and their families to face an unnecessarily prolonged hospitalization, and consequently reduces the availability of beds mainly in critical periods, such as winter season. It is essential to correct quickly the gap between the Northern, Central and Southern regions of Italy. This difference, as stated by De Curtis et al., indicates a need to strengthen pediatric care, by creating services that are currently not evenly distributed throughout the Italian territory [9].

To facilitate the activation of SSOs in hospitals, it is also necessary to guarantee adequate economic recognition for this form of assistance, particularly respectful of the needs of a child and his family. A reduced financial reward compared to the care commitment it entails, can help induce some hospitals to limit the activation of SSOs [10, 11].

Moreover, the single economic motivation is not enough to justify the delay in the adoption of guidelines, as 96,08%% SSOs are already active, compared to 40,00% of active SSOs in regions which have not yet adopted the guidelines.

We hope that the publication of these preliminary data will induce “reluctant” regions to modernize the pediatric emergency network, through a quick adoption of measures established from Status – Regions Conference of 1^st^ August 2019, of which SSO is only one of the main issues.

It is necessary that in Italy the best hospital welfare is guaranteed to minors, independently from the Region in which he/she lives.

## Data Availability

The datasets analysed during the current study are not available because the investigation is still ongoing, but they are available from the corresponding author on reasonable request.

## Abbreviations

SSO: Short-stay Observation
ED: Emergency Department
PH: Pediatric Hospital
GH: General Hospital with pediatric wards
FVG: Region of Friuli-Venezia-Giulia
SIP: Italian Society of Pediatrics
SIMEUP: Italian Society of Pediatric Emergency Medicine
SIPO: Italian Society of Hospital Pediatrician
DEA: Emergency and Triage Department (Dipartimento di Emergenza ed Accettazione)
